# Overview of Neuro-Ophthalmic Findings in Leukodystrophies

**DOI:** 10.3390/jcm13175114

**Published:** 2024-08-28

**Authors:** Charlotte Maria Bettinger, Simon Dulz, Yevgeniya Atiskova, Helena Guerreiro, Gerhard Schön, Philipp Guder, Sarah Lena Maier, Jonas Denecke, Annette E. Bley

**Affiliations:** 1 Children’s Hospital, University Medical Center Hamburg-Eppendorf, 20251 Hamburg, Germany; charlotte.bettinger@t-online.de (C.M.B.); p.guder@web.de (P.G.); sarah.lena.maier@gmail.com (S.L.M.); j.denecke@uke.de (J.D.); 2 Department of Ophthalmology, University Medical Center Hamburg-Eppendorf, 20251 Hamburg, Germany; 3 Department of Diagnostic and Interventional Neuroradiology, University Medical Center Hamburg-Eppendorf, 20251 Hamburg, Germany; h.desousa@uke.de; 4 Center of Experimental Medicine, Institute for Medical Biometry and Epidemiology, University Medical Center Hamburg-Eppendorf, 20251 Hamburg, Germany; g.schoen@uke.de

**Keywords:** leukodystrophy, white matter disorder, eye movement disorder, optic atrophy, neuro-ophthalmologic symptoms, visual impairment

## Abstract

**Background:** Leukodystrophies are a group of rare genetic diseases that primarily affect the white matter of the central nervous system. The broad spectrum of metabolic and pathological causes leads to manifestations at any age, most often in childhood and adolescence, and a variety of symptoms. Leukodystrophies are usually progressive, resulting in severe disabilities and premature death. Progressive visual impairment is a common symptom. Currently, no overview of the manifold neuro-ophthalmologic manifestations and visual impact of leukodystrophies exists. **Methods:** Data from 217 patients in the Hamburg leukodystrophy cohort were analyzed retrospectively for neuro-ophthalmologic manifestations, age of disease onset, and magnetic resonance imaging, visual evoked potential, and optical coherence tomography findings and were compared with data from the literature. **Results:** In total, 68% of the patients suffered from neuro-ophthalmologic symptoms, such as optic atrophy, visual neglect, strabismus, and nystagmus. Depending on the type of leukodystrophy, neuro-ophthalmologic symptoms occurred early or late during the course of the disease. Magnetic resonance imaging scans revealed pathologic alterations in the visual tract that were temporally correlated with symptoms. **Conclusions:** The first optical coherence tomography findings in Krabbe disease and metachromatic leukodystrophy allow retinal assessments. Comprehensive literature research supports the results of this first overview of neuro-ophthalmologic findings in leukodystrophies.

## 1. Introduction

Leukodystrophies (LDs) are a group of rare genetic neurodegenerative diseases characterized by a predominant involvement of the white matter of the central nervous system (CNS) [1,2,3]. All structural components of the white matter can be affected, including oligodendrocytes, astrocytes, microglia, axons, and blood vessels [3,4,5], resulting in dysfunctional synthesis and maintenance of myelin [1,6]. Hypomyelinating and demyelinating forms are distinguished according to myelin presentation over time [2,5,7].

Hypomyelinating leukodystrophies are usually caused by delayed and/or insufficient formation of myelin [2,8,9]. A typical hypomyelinating leukodystrophy is Pelizaeus–Merzbacher disease (PMD), and the appearance of the first symptoms occurs early after birth with developmental delay and nystagmus [2].

In demyelinating leukodystrophies, normal appearing myelin formation is followed by a loss of myelin, which leads to symptoms [2,10]. Classic demyelinating leukodystrophies are metachromatic leukodystrophy (MLD) and Krabbe disease (KD). In MLD, reduced activity of the enzyme arylsulfatase A leads to an accumulation of sulfatides in the central and peripheral nervous system, resulting in demyelination [11,12].

Clinical onset may occur at any age, from prenatal life to senescence [5,7], although many forms begin in infancy and affect clinically inconspicuous children until symptom onset [5,7]. Leukodystrophies are caused by different underlying pathomechanisms and lead to a broad spectrum of clinical symptoms. The clinical course is usually progressive, with extensive loss of motor and mental abilities, often leading to helplessness and premature death [1,2]. Initial symptoms often include an arrest of motor or cognitive development followed by further deterioration and regression. The most common neurological findings include motor deficits, which often manifest with a delay in reaching motor milestones [6,13,14]. Cognition is often affected during the course of the disease, especially in older patients, but deterioration of cognitive functions may also occur at disease onset. However, milder forms have also been described and are characterized by longer intervals of symptom stability or even improvement [2]. Most leukodystrophies are inherited in an autosomal recessive manner [6,15], although other modes of inheritance exist (autosomal dominant or X-linked) [15].

Neurodegenerative diseases are often associated with ocular and optic tract involvement [16,17,18,19]. Various manifestations range from the anterior eye, as noted in patients with cerebrotendinous xanthomatosis (CTX), who develop cataracts [20] via myopia in 4H syndrome to optic atrophy in various leukodystrophies [21].

Although ocular involvement has been described in numerous single reports of leukodystrophies [15], no broad observation of a large cohort of multiple leukodystrophies has been attempted thus far. A better understanding of visual impairment in leukodystrophy patients may have an impact on the clinical understanding of these diseases, may even facilitate earlier diagnosis [15], and may help to identify novel outcome measures in clinical trials.

Herein, we analyzed different neuro-ophthalmologic symptoms (NOSs) and correlated them with the presence of leukodystrophy and findings in the brain on MRI. We show the first findings of retinal nerve fiber layer imaging (RNFL) performed by optical coherence tomography (OCT) and the potential of this noninvasive imaging method for quantifying extracerebral neuronal tissue.

## 2. Materials and Methods

### 2.1. Data Extraction

The data of 349 patients with leukodystrophies were screened. The data of 217 patients with genetically and/or biochemically confirmed diagnoses of leukodystrophy from the Hamburg leukodystrophy cohort (HLC), Department of Pediatrics at the University Medical Center Hamburg-Eppendorf, Germany, who met the data protection criteria were analyzed retrospectively (informed consent according to PV3782, Ethics Committee of the Medical Association of Hamburg, Hamburg, Germany). The analyzed data records from international as well as German patients were anonymized and processed according to applicable regulations. The defined secondary outcome variables were age, sex, age at symptom onset, type of leukodystrophy, and survival.

For X-linked adrenoleukodystrophy (X-ALD) patients, only male patients of all ages were analyzed. Females with X-ALD were listed as others. For some analyses, LDs were grouped into demyelinating and hypomyelinating LDs. The following LDs were identified under demyelinating conditions: MLD, X-ALD, KD, Canavan disease (CD), Alexander disease (AxD), vanishing white matter disease (VWM), Aicardi–Goutières syndrome (AGS), and megalencephalic leukoencephalopathy with subcortical cysts (MLC). Under hypomyelinating LDs, we summarized the following: PMD and other less frequent leukodystrophies, such as 4H syndrome and X-linked hypomyelination with spondylometaphyseal dysplasia (H-SMD).

Clinical status was assessed using a retrospective review of neurological evaluations, neurodevelopmental assessments, and ophthalmological evaluations. The following neuro-ophthalmologic symptoms were defined as primary outcome variables, including the onset of the different neuro-ophthalmologic symptoms:

- visual function (e.g., reduced visual acuity, contrast sensitivity, color discrimination, peripheral vision, and higher order visual processing);
- eye movement disorders (EMD) (e.g., nystagmus, strabismus that was defined as any heterotropia at near and/or distance fixation);
- fixational eye movement disorders (FEMD) (e.g., no ability to fix and follow or track objects);
- pupillary light reflex (defined as either absent, delayed, or slowed); and
- anterior and periocular segment findings (e.g., cataracts, ptosis, infections of the eye) and posterior segment findings (e.g., optic atrophy).

Normal as well as pathologic results were recorded.

### 2.2. MRI Analysis

MRI scans of patients with pathological neuroophthalmological findings were analyzed within a maximum of 1.5 years before or after the reported onset of neuro-ophthalmologic symptoms. T1W and T2W images as well as fluid-attenuated inversion recovery (FLAIR) images in the transverse and sagittal planes were used for the analysis. Brain MR images were acquired at different centers using numerous different scanners and distinct imaging protocols. Every examination included basic MRI sequences, which were used for review. Two independent experienced raters (HG and AB) evaluated these sequences with special attention to the visual tract, which consists of the optic nerve, the optic chiasm, the optic tract, the lateral geniculate nucleus, and the visual cortex in the occipital lobes [22]. Signal abnormalities were identified and recorded.

### 2.3. Optical Coherence Tomography and Retinal Nerve Fiber Measurement

OCT analysis was performed with a swept-source optical coherence tomography (SS-OCT) instrument (Topcon DRI OCT Triton Plus, Topcon Medical Inc., Tokyo, Japan). A circular peripapillary scan was performed in each eye, and retinal nerve fiber (RNFL) measurements (μm) were extracted from ImageNet 6 software (Topcon Medical Inc., Tokyo, Japan). One experienced rater (SD) evaluated the available OCT scans.

### 2.4. Visual Evoked Potential (VEP)

The results of the VEP examinations of the patients’ records were analyzed. If VEP was performed outside of our clinic, no information on the examination protocol was available. VEP findings were recorded and classified as “pathological” or “normal” without further specification. For simplification a pathological VEP was considered as neuro-ophthalmologic symptom. VEP examinations that were performed at our institution were performed using RetiPort (Roland Consult Stasche & Finger GmbH, Brandenburg an der Havel, Germany). For the device settings for pattern reversal VEP, the screen illumination was set to 50 cd/m^2^, the contrast was set to 100%, the pattern turnover ratio was set to 2/sec, and the sweep time was set to 300 ms. For the filter settings, the subfilter was set to 1 Hz. In the case of flash VEP, a 3 cd/s/m^−2^ flash stimulus with a flash rate of 1 Hz was used. All electrodes were applied according to the ISCEV guidelines [23].

### 2.5. Literature Review

To compare our data with data from the literature, a systematic analysis of the literature was conducted. The search strategy for the biomedical database MEDLINE via PubMed used a combination of key words (MeSH-terms) and free text (last searched May 15, 2022; the search strategy was as follows: ((“Leukodystrophy”[MeSh]) OR (“Leukoencephalopathies” [MeSh]) OR (“MLD”) OR (“Leukodystrophy, Metachromatic” [MeSh]) OR (“Canavan disease” [MeSh]) OR (“Krabbe disease” [MeSh]) OR (“Leukodystrophy, Globoid Cell” [MeSh]) OR (“Adrenoleukodystrophy” [MeSh]) OR (“ALD”) OR (“PMD”) OR (“Pelizaeus–Merzbacher disease” [MeSh]) OR (“VWM”) OR (“Vanishing white matter”) OR (“Alexander disease” [MeSh]) OR (“Aicardi–Goutières syndrome” [MeSh]) OR (“AGS”) OR (“MLC”) OR (“Megaleukoencephalopathy” [MeSh])) AND ((“Nystagmus, Pathologic”[MeSh]) OR (“Nystagmus, Congenital” [MeSh]) OR (“Optic Atrophy” [MeSh]) OR (“Strabismus” [MeSh]) OR (“Visual inattention”) OR (“Pupil disorders” [MeSh]) OR (“Eye movement” [MeSh]) OR (“Fixation, Ocular” [MeSh]) OR (“Visual Fields” [MeSh]) OR (“Vision, Ocular” [MeSh]) OR (“Pupillary reaction”) OR (“Pupillary response”) OR (“VEP”) OR (“Visual evoked potentials” [MeSh])).

**Figure 1:**
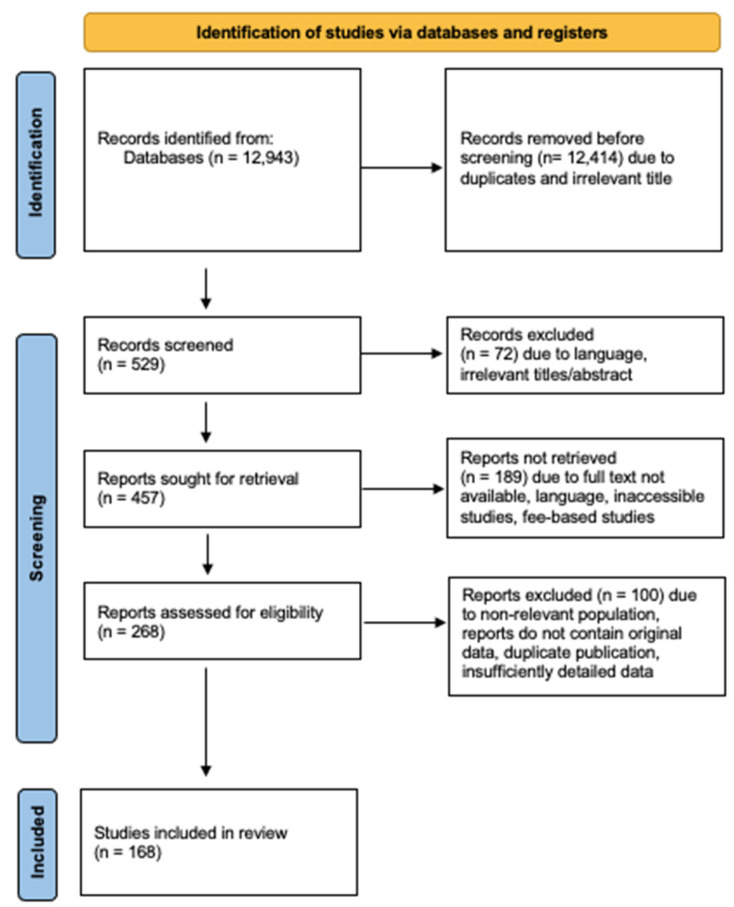
PRISMA 2020 flow diagram. Source: [134].

The following databases were searched using free text combinations: EMBASE, ISI Web of Knowledge, Cochrane Central Register of Controlled Trials (CENTRAL), BioMed Central, and ScienceDirect (last searched 15 May 2022). No language restrictions were imposed. We additionally screened clinical trial registries, including WHO International Clinical Trials Registry and ClinicalTrials.gov, as well as the reference lists of the full texts of potentially relevant studies and review articles (last searched 15 May 2021). We searched for prospective and retrospective longitudinal observational studies investigating the association between leukodystrophies and the neuro-ophthalmologic symptoms. We also analyzed reviews, editorials, and commentaries. CB screened the identified articles and reviewed the full texts of the potentially relevant references. CB performed the data extraction and analysis. All references were managed and stored in EndNote 20 [24,25,26,27,28,29,30,31,32,33,34,35,36,37,38,39,40,41,42,43,44,45,46,47,48,49,50,51,52,53,54,55,56,57,58,59,60,61,62,63,64,65,66,67,68,69,70,71,72,73,74,75,76,77,78,79,80,81,82,83,84,85,86,87,88,89,90,91,92,93,94,95,96,97,98,99,100,101,102,103,104,105,106,107,108,109,110,111,112,113,114,115,116,117,118,119,120,121,122,123,124,125,126,127,128,129,130,131,132]. The literature search (Lit.) identified 529 possibly relevant references. Of these, 268 references were included for full-text evaluation. In total, we incorporated data from 168 publications into the analysis. Figure 1 provides an overview of the study selection process according to the PRISMA guidelines [133]. The flowchart shows the number of studies identified, excluded, and included, as well as the reasons for the exclusions.

### 2.6. Data Analysis

The data were analyzed in a Microsoft Excel Version 16.77 (23091003) spreadsheet. As descriptive statistics, arithmetic means and standard deviations are reported for the metric characteristics and counts, and proportions are reported for categorical variables.

For the analysis of time to symptom onset (FEMD, EMD, and visual function), the time between diagnosis and symptom onset was used. For patients without symptoms, the time between diagnosis and the last hospital visit was used. Statistical analyses were performed by the UKE Institute of Biometry and Statistics (GS) using the program R¹ (Version 3.4.3).

## 3. Results

### 3.1. Patient Characteristics

The data of 217 patients with various leukodystrophies in the Hamburg leukodystrophy cohort (HLC) and 1153 patients from the literature search (LCC) were analyzed. The median age of disease onset of HLC patients was 7 months, varying from leukodystrophies with an early onset of 1 month as noted for PMD to leukodystrophies with a later onset as noted for X-ALD (median age of onset of 94 months). In the literature, the median age of onset of the disease was 6 months, ranging from 2 months for PMD to 83 months for X-ALD. A balanced sex distribution was observed in both cohorts, excluding patients with X-linked inherited leukodystrophies such as PMD, X-ALD, and H-SMD. Neuro-ophthalmologic symptoms occurred in 68% of HLC and 76% of LLC patients during the course of the disease. The occurrence of neuro-ophthalmologic symptoms depended on the type of leukodystrophy. In the HLC, patients with PMD (100%) and CD (100%) were most frequently affected by neuro-ophthalmologic symptoms, a tendency that was also observed in the LLC (PMD 98%, CD 83%). HLC patients with AxD (30%) and MLD (42%) were less frequently affected by neuro-ophthalmologic symptoms. In the LLC, the lowest prevalence was identified in patients with VWM (29%) and MLD (32%). Most neuro-ophthalmologic findings were progressive in patients that were observed over an extended period. Follow up time spans were 0–39 years with a median of 4.2 years (some patients only presented to us once).

An overview of the age at onset of the disease, the occurrence of neuro-ophthalmologic symptoms, and patient sex for different leukodystrophies is shown in Table 1.

### 3.2. Neuro-Ophthalmologic Disease Manifestations

The majority of leukodystrophy patients (68%) developed one or more than one (total sum of 366 neuro-ophthalmologic findings in the HLC) neuro-ophthalmologic symptoms during the course of the disease. Neuro-ophthalmologic symptoms occur early or late during the course of the disease. On average, if a leukodystrophy patient became symptomatic, the first description of neuro-ophthalmologic symptoms occurred within 6 months (range from 0 months to 33 years). Neuro-ophthalmologic symptoms were reported to be the first symptom of leukodystrophy in 26% of HLC patients (*n* = 38). In 8% (*n* = 12) of the patients, neuro-ophthalmologic symptoms were even reported to be present at birth, mainly in patients with PMD (*n* = 7) and CD (*n* = 4). The first neuro-ophthalmologic symptoms were reported to occur in the same month as disease onset in PMD and one month after disease onset in CD. Patients with AGS or KD were reported to suffer from neuro-ophthalmologic symptoms early in the course of the disease (4 and 5 months after disease onset, respectively). In those with X-ALD, neuro-ophthalmologic symptoms occurred 9 months after the onset of the disease. However, in other leukodystrophies, neuro-ophthalmologic symptoms were reported to occur rather late during the course of the disease, as were MLD (median 31 months), AxD (median 44 months), VWM (median 86 months) and MLC (median 100 months).

We differentiated neuro-ophthalmologic symptoms in all leukodystrophies by the kind of neuro-ophthalmic symptom. The most common were eye movement disorders (EMDs) in 30% and fixational eye movement disorders (FEMDs) in 15%, followed by posterior segment findings in 13%. The frequencies of all analyzed neuro-ophthalmologic symptoms are listed in Table 2.

In total, 1057 pathological findings were extracted from 1153 patients in the literature (LLC). As noted in the HLC, the most common findings were eye movement disorders (60%), followed by impaired visual function (21%) and posterior segment findings such as optic atrophy (14%), as shown in Table 2.

The neuro-ophthalmologic symptoms were differentiated according to the type of leukodystrophy and the type of the neuro-ophthalmologic symptom. Frequencies of specific neuro-ophthalmologic symptoms in the HLC and LLC are shown in Table 3.

#### 3.2.1. EMD Nystagmus as an Early Sign of Hypomyelinating Leukodystrophies

The eye movement disorder nystagmus was the most common neuro-ophthalmologic symptoms in our analyzed leukodystrophy patients (28%). It occurred most frequently in PMD patients (100%), followed by CD patients (62%). The frequency of nystagmus is shown in Table 3. In 28% of patients, nystagmus was the first clinical sign of disease, and it was congenital in 18%. Considering only hypomyelinating forms of leukodystrophies (*n* = 26), nystagmus occurred in 77% of patients with a median of 0 months after disease onset (range 0 months to 20 years). In the demyelinating group (*n* = 191), only 21% of patients exhibited nystagmus with a median of 5 months after disease onset (range 0 months to 13 years). In 22% of patients, the nystagmus disappeared completely during the course of the disease, with a median age of 29 months (range 7 months to 15 years) after the first onset of nystagmus, regardless of the type of leukodystrophy. For 72% (569/790) of all LLC patients, nystagmus was reported. Among patients with nystagmus, 86% had a hypomyelinating form of leukodystrophy, and 14% had a demyelinating form of leukodystrophy.

#### 3.2.2. EMD Strabismus

Strabismus was diagnosed in 18% of HLC patients, with a median age of onset of 8 months (range 0 months to 33 years) after disease onset. Patients with PMD (31%) were affected most frequently, followed by those with CD (26%) and other LDs, as shown in Table 3. In terms of hypomyelinating forms, 42% of patients showed strabismus with a median time span of 12 months after disease onset. In terms of demyelinating forms, 15% of patients developed strabismus with a median time span of 6 months after disease onset. Within the LLC, a total of 34% (68/202) of patients developed strabismus. The distribution of strabismus in the different forms of leukodystrophies is listed in Table 3. Among these patients who developed a strabismus, 9% had hypomyelination, and 91% had a demyelinating form of leukodystrophy.

#### 3.2.3. Fixational Eye Movement Disorders (FEMD)

Twenty-five percent of HLC patients developed fixational eye movement disorders, with a median age of onset of 7 months (range 0 to 326) after disease onset. Patients with CD (56%) and AGS (44%) were affected most commonly. In LCC, 69% (24/35) of the patients were described to have a fixational eye movement disorder (further details in Table 3).

#### 3.2.4. Visual Function

Impaired visual function was observed in 11% of all HLC patients, with a median age of onset of 18 months (range 0 to 432 months) after disease onset. Visual function impairment was most commonly reported in X-ALD (27%) and CD (21%) and less frequently in VWM (8%) and other forms of leukodystrophies (further details in Table 3). In the LLC, 53% (223/417) of patients had a deficit in visual function. In detail, of the patients who showed impaired visual function, 63% were patients with demyelinating leukodystrophies, and 29% were patients with hypomyelinating leukodystrophies.

#### 3.2.5. Pupillary Light Reflex

A pathologic pupillary light reflex was less frequently reported in 14% (*n* = 31) of HLC patients, without major differences in patients with demyelination or hypomyelination. Median age of onset was 35 months (range 0 to 284) after disease onset. In the LLC, 39% (*n* = 28/72) were found to have a pathological light reaction. Further details of the occurrence of a pathological pupillary light reflex in the different forms of leukodystrophies of both cohorts are depicted in Table 3.

#### 3.2.6. Anterior and Periocular Segment Findings

In the HLC, 11% (*n* = 23) had pathological findings of the anterior or periocular segment. The findings were inflammation of the anterior or periocular segment (hordeolum, blepharitis, and conjunctivitis) in 30% (*n* = 7) most frequently in patients with CD (*n* = 3) and MLD (*n* = 2), cataracts in 30% (*n* = 7) of patients with CTX (*n* = 2) and HCC (*n* = 1), and ptosis in 22% (*n* = 5) of patients with MLD (*n* = 2). Sparse LCC data did not allow for sufficient analysis.

#### 3.2.7. Posterior Segment Findings as Optic Atrophy

Only 14% of HLC patients (*n* = 30) were reported to develop optic atrophy during the progression of the disease, with a median age of onset of 25 months (range 0 to 319 months) after disease onset. Patients with AGS (33%; *n* = 3/9) and PMD (31%, *n* = 4/13) were affected most frequently, followed by those with other LDs, as shown in Supplemental Table S1. Patients with hypomyelinating forms (35%; *n* = 9/26) were more often affected than patients with demyelinating forms (11%; *n* = 21/191). Among all patients identified by the literature search, 38% (145/385) developed optic atrophy during the course of the disease. Overall, 14% of patients with optic atrophy had hypomyelinating forms of leukodystrophy, and 86% had demyelinating forms of leukodystrophy.

### 3.3. VEP Results in Leukodystrophies

In total, 57 VEP results in 54 HLC symptomatic and pre-symptomatic patients were reported. Thirty-five patients showed pathological VEP findings (65%). A detailed analysis of all LD forms and percentages of pathologic VEP findings is shown in Table 4. In the group with hypomyelinating forms of leukodystrophies, pathological VEP findings were noted in 100% (*n* = 8/8) of patients, and in the group with demyelinating forms of leukodystrophies, it was found in 59% (*n* = 27/46) of patients. Patients exhibiting normal VEP findings had a median illness duration of 15 months, whereas those with pathological VEP findings demonstrated a significantly longer median illness duration of 26 months.

Almost all 35 patients with pathological VEP findings had at least one other neuro-ophthalmologic symptom. In total, the 35 patients with pathological VEP findings had 87 additional neuro-ophthalmologic findings. The most common were EMD (*n* = 29), followed by posterior segment findings (*n* = 16) and FEMD (*n* = 10). Normal VEP findings were initially observed in 22 patients. Three patients (1 MLD, 1 AxD, 1 MLC) developed pathological VEP findings during the course of the disease, leaving 19 patients with normal VEP findings during the observation of AxD (*n* = 2/3), KD (*n* = 3/4), X-ALD (*n* = 3/5), VWM (*n* = 2/4), and MLD (*n* = 8/19) patients. Patients with normal VEP findings had a median disease duration of 15 months.

In LLC patients, 66% (*n* = 52/79) had pathologic VEP results. Pathological VEP results occurred more frequently in hypomyelinating forms of leukodystrophy (83%; *n* = 15/18) than in demyelinating forms (61%; *n* = 37/61). More details are shown in Table 4.

### 3.4. OCT in Leukodystrophies

**Figure 2:**
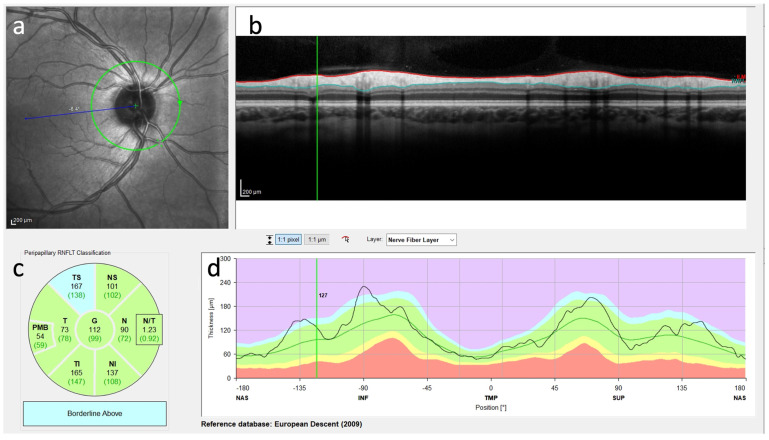
Diagrammatic map revealing the OCT examination of a patient with metachromatic leukodystrophy, showing regular peripapillary retinal nerve fiber layer (RNFL) thickness in the right eye measured by spectral domain OCT (SD-OCT). (**a**) The appearance of the right optic nerve. (**b**) Transverse image of the RNFL. (**c**) Segmentation of the RNFL. Each area is colour coded to indicate whether the thickness of the nerve fiber layer is in the normal range (green) or out of range (yellow or red). (**d**) RNFL thickness map of the right eye. The different coloured areas in the background represent reference areas (normal distributions). The black line represents the measured thickness of the patient’s nerve fiber layer. This curve can be used to recognize whether the thickness in certain areas is within (green area) or outside (red area) the normal range.

OCT was performed in 10 leukodystrophy patients (3 KD, 2 MLD, 2 X-ALD, 1 MLC, 1 4H syndrome, 1 CTX). The findings of nine patients were available for evaluation. Three patients showed abnormal thinning of the retinal nerve fiber layer on OCT, and six showed no abnormalities. The six patients with normal OCT findings (Figure 2, normal findings in a–d) were reported to be either early in their course of the disease or were suffering from a leukodystrophy with a late onset. One of the MLD patients has received an experimental treatment and has been symptom stable for years, and the one patient with proven X-ALD is presymptomatic for ccALD suffering only from Addison’s disease.

**Figure 3:**
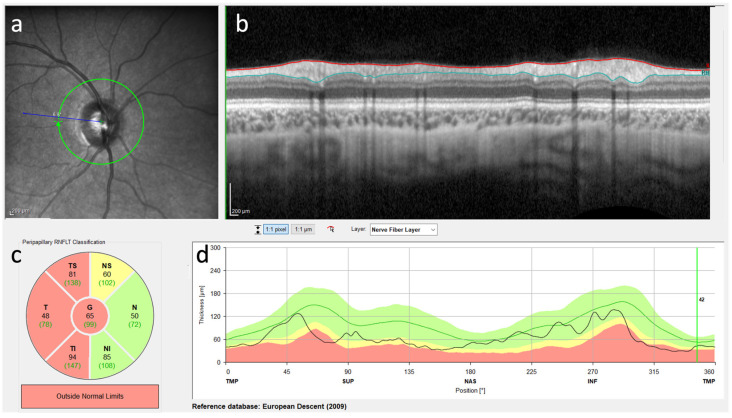
Diagrammatic map revealing the OCT examination of a patient with Krabbe disease, showing superior RNFL thinning in the right eye. (**a**) The appearance of the right optic nerve. (**b**) Transverse image of the RNFL. (**c**) Segmentation of the RNFL. Each area is colour coded to indicate whether the thickness of the nerve fiber layer is in the normal range (green) or out of range (yellow or red). (**d**) RNFL thickness map of the right eye. The different coloured areas in the background represent reference areas (normal distributions). The black line represents the measured thickness of the patient’s nerve fiber layer. This curve can be used to recognize whether the thickness in certain areas is within (green area) or outside (red area) the normal range.

Two patients with KD and one patient with 4H syndrome showed abnormal thinning of the retinal nerve fiber layer (RNFL) on OCT (Figure 3). At the time of the OCT examination, optic atrophy was present in all three patients, which was diagnosed by inspection by an ophthalmologist. Neuro-ophthalmologic symptoms were detected in almost all patients with normal OCT results; in detail, two had cataracts, and three had refractive errors.

### 3.5. MRI Findings Correlate with Neuro-Ophthalmologic Findings

We analyzed 49 MRI scans of 49 patients for whom cranial MRI was performed 1.5 years before or after the onset of neuro-ophthalmologic symptoms. Patients were affected by the following neuro-ophthalmologic symptoms: EMD (*n* = 23), FEMD (*n* = 29), and pathological pupillary light reflex (*n* = 2). Five patients had two neuro-ophthalmologic symptoms at the time of evaluation. In-depth analysis of the visual tract was performed on T1W, T2W, and FLAIR cranial MR images.

**Figure 4:**
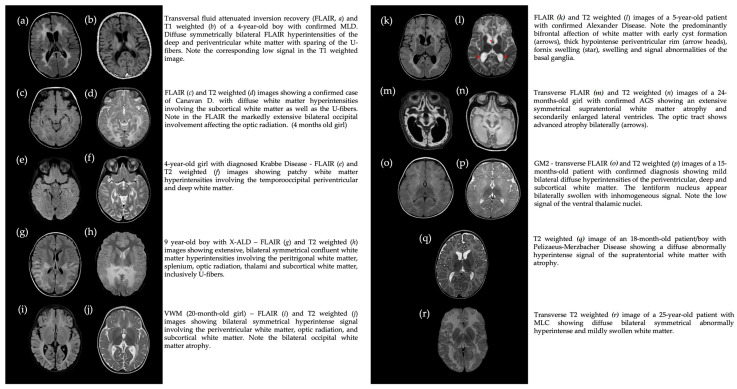
Pathological brain MRI findings of selected patients with myelin involvement of the visual pathway.

The majority (*n* = 47/49) exhibited pathologic visual pathway effects on the brain (Figure 4), with effects on the periventricular parietooccipital white matter. Only two patients presented with age-appropriate MR images. Symptoms of these patients were a transient setting-sun phenomenon in an otherwise presymptomatic KD and strabismus in an X-ALD female conductor.

**Figure 5:**
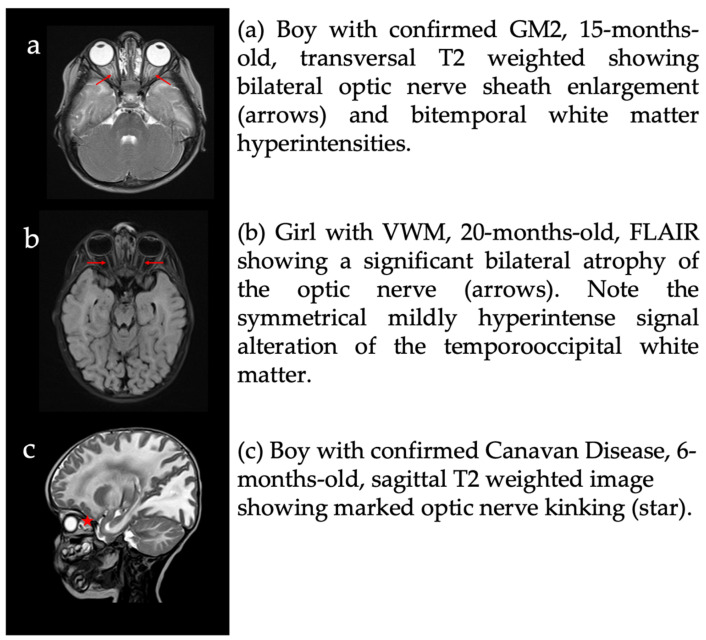
Pathological findings of the optic nerves.

Myelin involvement of the parieto-occipital white matter and visual pathway was observed in all MRI images. Additional distinct findings were optic nerve kinking (increased tortuosity of the optic nerve) in eight patients (3 CD, 1 KD, 1 PMD, 1 X-ALD, 1 4H-syndrome, 1 Col4a1), optic nerve sheath dilatation/optic disc swelling in seven patients (1 CD, 3 KD, 1 VWM, 1 4H syndrome, 1 GM2) and optic atrophy in one patient (VWM), as shown in Figure 5.

## 4. Discussion

This study describes the occurrence and frequency of different neuro-ophthalmologic symptoms in 217 patients with different leukodystrophies in a leukodystrophy cohort and compares them with data from 1153 patients reported in the literature. We further report the heterogeneity of the occurrence of the neuro-ophthalmologic symptoms in relation to the timing of the first disease symptom in patients with different types of leukodystrophies. We describe the corresponding findings of ophthalmologic and other diagnostic methods, such as MRI, VEP, and OCT. In two patients with Krabbe disease and one patient with 4H syndrome, we report a previously unpublished abnormal thinning of the retinal nerve fiber layer observed in OCT imaging.

Neuro-ophthalmologic symptoms are common findings in leukodystrophies. In our cohort, 68% of our patients were reported to suffer from neuro-ophthalmologic symptoms, compared to 76% in the literature cohort. The occurrence of neuro-ophthalmologic symptoms was 100% in patients with PMD and CD in our cohort and was also very frequent feature in patients in the literature with PMD (98%) and CD (83%). In 26% of our cohort, neuro-ophthalmologic symptoms were among the first symptoms of the disease, especially in patients suffering from PMD and CD. Hence, the majority of neuro-ophthalmologic symptoms occurs secondarily during the course of the disease. Some symptoms occurred usually earlier in the course of the disease, such as EMD (median nystagmus 0 months and strabismus 8 months after disease onset) and FEMD (median 7 months after disease onset). Other neuro-ophthalmologic symptoms occurred later during the course of the disease as optic atrophy occurred at a median of 25 months after disease onset and pathologic light response at a median of 35 months after disease onset. These finding may support development of specific treatments according to affected functions during the course of the disease.

Interestingly, we found that the most common neuro-ophthalmologic symptoms in leukodystrophy patients (30% in our cohort, 60% in the literature) were eye movement disorders. Among these patients, nystagmus was most common (54%) and appeared at disease onset in our cohort, predominantly in patients with hypomyelinating leukodystrophies, including PMD, as reported previously [13]. Nystagmus in general, as an eye movement disorder, may reflect the interruption of visual input, which is important for optimal eye movement [135]. It is frequently observed in children with cortical and subcortical visual disorders and might be associated with severe visual disturbances and inability to fix and track objects [136]. Nystagmus is not solely related to neuro-ophthalmic pathologies, but it can also be observed in diseases of the vestibular apparatus or central nervous regions such as those of the brainstem [137] and as a sequel of intoxications and metabolic disorders [138]. It is not yet understood why nystagmus occurs predominantly in hypomyelinating forms of leukodystrophies. It is possible that the disruption of early myelinating structures (as for example infratentorial structures) might be related to the higher occurrence.

The optic tract crosses the brain and is dependent on the degree of myelination. In our patients with hypomyelinating leukodystrophies, 100% of the VEP findings were abnormal, and 83% of the VEP findings in the literature group with hypomyelinating leukodystrophies were abnormal, reflecting insufficient myelination of the optical tract/optic pathway. The occurrence of nystagmus in 100% of our PMD patients appears conclusive with this finding. Interestingly, none of our PMD patients reported suffering from a visual function deficit. Since VEPs reflect the sum of the functions of the retina, the visual pathway, and the processing of visual stimuli by the brain, these findings mainly represent a pathology of the visual pathway or visual cortex in LD [139]. We hypothesize that nearly all patients with leukodystrophy exhibit pathological VEP during the progression of the illness. Due to their age and disabilities, these patients are usually unable to communicate visual symptoms or participate in standardized tests, so the VEP serves as a surrogate parameter for the duration or stage of the disease [140].

It is also not understood why, regardless of whether it is a hypomyelination or a demyelinating form of leukodystrophy, in 22% of HLC patients, the nystagmus disappeared completely over the course of the disease, with a median of 29 months (range 7 months to 15 years) after onset. Patients with hypomyelinating leukodystrophies, such as PMD or 4H syndrome, often gain further developmental milestones over time before a regression is noted. Atrophy is often described to occur after some time, often years, in hypomyelinating leukodystrophies, such as PMD [141] We assume that this partial, at least functional, maturation may also affect the nystagmus positively.

In demyelinating leukodystrophies, we rather assume that the initial affection of structures causes neuronal imbalances that lead to the nystagmus. Further degeneration with almost complete loss of myelinated structures and progressive atrophy may affect the symptoms of nystagmus, leading to blindness with regression of nystagmus due to more unspecific oculomotor problems. Further research with prospective assessments, such as ophthalmologic examination, MRI, and VEP, may support elucidation of the pathogenesis of nystagmus in different LDs and in general.

The second most common eye movement disorder in our cohort was strabismus (18%), with a median onset of 8 months after disease onset. In our cohort, strabismus was most common in hypomyelinating LDs, as in PMD. Forty-two percent of patients showed strabismus with a median time span of 12 months after disease onset. However, in demyelinating forms, 15% of patients developed strabismus with a median time span of 6 months after disease onset. A specific correlation to one or more of the involved neurologic systems that are important for eye movement coordination in the brainstem, such as cranial nerve nuclei, the supraoculomotor area, the medial longitudinal fasciculus, and paramedian pontine formation [142], was not possible according to our retrospective MRI analyses. In the literature cohort, 34% of patients developed strabismus. Among these patients, 9% had a hypomyelinating form of leukodystrophy, and 91% had a demyelinating form of leukodystrophy.

Visual function deficits in patients with leukodystrophies have also been reported but are less common, with only 11% in our cohort and 53% in the literature. We assume that the composition of our cohort with a greater percentage of patients in their early disease course might explain this discrepancy as well as the method used for the literature search, as an association between leukodystrophy and visual deficits was explicitly sought.

Abnormalities in pupillary light reflexes according to clinical examination were less frequent in our cohort (14%) than in the literature cohort (39%). As abnormal myelination usually affects the nerve conduction velocity, we assume that subtle changes in the reaction time of pupillary light reflexes might be persistent but would need more sensitive methods, such as pupillometry. This topic is subject to further research. 

Pathologic findings of the anterior and periocular segments occurred in 11% of our cohort. Inflammation, such as blepharitis, conjunctivitis, or hordoleum, was most common in patients with Canavan disease (42%, *n* = 3/7). Prospective studies are necessary to reveal potential causes, such as reduced blinking frequencies, in children with CD. As previously published, cataracts were present in patients with CTX [20].

Analysis of 54 patients with VEP revealed a pathologic finding in the majority of the reported examinations (65%), which is in line with the results in the LCC where 66% also showed pathological VEP findings (52 of 79 patients). When grouped by hypomyelinating and demyelinating forms of leukodystrophies, pathological VEP findings were noted in 100% (*n* = 8/8) of our cohort in the group with hypomyelinating leukodystrophies (83% in LCC) and in 59% (*n* = 27/46) of our cohort in the group with demyelinating forms of leukodystrophies (61% in LCC).

Involvement of the optic nerve during disease progression is a known feature of leukodystrophies. As an example in MLD, histopathological findings have revealed metachromatic deposits in retinal ganglion cells, optic nerves, and ciliary nerves [143]. Optic atrophy is mainly described later during the disease course [2]. We believe the normal VEP results in 19 patients can be attributed to the early timing of the examinations, conducted at a median of 15 months after disease onset.

The discrepancies in the frequency of ophthalmologic findings between HLC and LLC data might be explained by the selection of studies or articles based on predefined search criteria and keywords. There might be an overestimation of the incidence in the literature as reported cases are often more severely affected or have a longer follow-up time. In our cohort, there are many cases who are still early in the course of the disease, for whom we suspect that neuro-ophthalmologic symptoms may still occur during the course of the disease. A follow-up would be useful and would provide information about the actual differences in frequency. A prospective systematic evaluation based on research would allow a significant reduction in this bias.

In our study, the OCT data of 9 patients with leukodystrophy were analyzed. Six patients with MLD (*n* = 2), X-ALD (*n* = 2), CTX (*n* = 1), or MLC (*n* = 1) and normal OCT findings were not in an advanced stage of the disease or suffered from a slowly progressive form of leukodystrophy. Three patients, two with KD and one with 4H syndrome, showed abnormal thinning of the retinal nerve fiber layer (RNFL). All patients with pathologic OCT presented at a progressed stage of disease and presented optic atrophy when OCT was performed. Interestingly, no reports of abnormal RNFL thinning in Krabbe disease or 4H syndrome patients, including one adult Krabbe patient without abnormal OCT findings [144], have been published to date. As the thickness of the RNFL is thought to reflect the integrity of the unmyelinated axons of the anterior visual pathway and the CNS white matter, future studies need to analyze the impact of OCT as a surrogate marker for disease progression. We did not observe hyperreflective retinal spots in the three pathologic OCT results of Krabbe and 4H syndrome patients, which are thought to be related to microglia activation [145]. Due to the limited number of patients who underwent OCT, we assumed that retinal changes might correlate with the degree of progression reflecting later stages of the disease. Further research should evaluate retinal involvement in leukodystrophies using OCT. This may impact decisions in the development of new treatments as not all application routes for CNS treatments might have effects on the retina.

MRI provides information on the extent and cause of white matter involvement in the brain [15,16,17]. In general, white matter involvement in LDs is bilateral and symmetric [146]. Characteristic MRI patterns are known for some LDs, which may contribute to the correct diagnosis [15]. For example, MLD patients show a distinct pattern of demyelination, which is also called the “stripe sign” or the “leopard skin sign” with stripes and patches [147]. There is also a common MRI pattern in X-ALD patients with symmetrical involvement of the parieto-occipital or frontal white matter and the adjacent part of the corpus callosum [146]. 

As we analyzed cerebral MRI within +/− 1.5 years before or after the reported onset of neuro-ophthalmologic symptoms, most MRIs revealed abnormal findings of the visual tract in the HLC. Most commonly, bilateral demyelination of the occipital white matter was found, whereas abnormalities of the optic nerves were second most common.

Eight patients showed optic nerve kinking on MRI, which, to our knowledge, has not been previously described in leukodystrophies. These patients suffer from CD, KD, PMD, X-ALD, 4H syndrome, and Col4a1. In the literature, optic nerve kinking has for example been reported in optic nerve gliomas, neurofibromatosis 1 [148,149], and aneurysms [150]. Seiff et al. cited the high water content in myxomatous tissue as a possible explanation for kink formation [151]. The pathophysiology of optic nerve kinking in LDs has yet be solved and may be the focus of future MRI studies. The small number of patients with an MRI analysis may affect the generalizability of the results. The selection criteria were independent of leukodystrophy, gender, age of onset, and disease severity. We therefore assume that this group represents our cohort well.

However, this study has limitations given the relatively small sample size of the individual leukodystrophies. In addition, data collection was only retrospective, and the length and intervals of the follow-up periods varies considerably. Reports of neuro-ophthalmologic and other clinical symptoms were part of the clinical examinations and not performed on a systematic prospective basis. Currently, for most leukodystrophies, there is no curative treatment available. As palliative care is the main treatment for most patients, complete and regular assessments with MRI, VEP, OCT and ophthalmologic examinations are not done as part of routine clinical care given the health system costs and patient burden. Systematic prospective studies within a research setting would allow a deeper understanding of NOS in leukodystrophies and may allow more targeted development of treatments.

Further follow-up studies should investigate the occurrence of neuro-ophthalmologic symptoms and the possible improvement of existing symptoms over a longer period of time with or without therapy. This is of interest as the evaluation of individual therapies is still discussed and the development of new treatment approaches is ongoing.

It must be taken into account that the disease stage of LD is important for the pathology of the optical tract, particularly in the case of demyelinating LD. The results therefore represent a longitudinal section of the course of the disease after diagnosis and should not be used as absolute frequencies of occurrence in the corresponding diseases. The collection of these data in the respective final stage of the diseases would therefore be expected to be associated with considerably higher figures.

## 5. Conclusions

Leukodystrophies remain one of the most devastating inherited neurological disorders in children and young adults. Our results demonstrate the importance of ophthalmic assessments as neuro-ophthalmologic symptoms are common in leukodystrophies and contribute to the burden of disease. An MRI scan of the brain is an appropriate tool to reveal pathologic alterations in the visual tract that are temporally correlated with symptoms. The first OCT findings in Krabbe and 4H syndrome patients demonstrate retinal findings. Further research needs to evaluate occurrence of retinal involvement in leukodystrophies.

## Figures and Tables

**Table 1:** Occurrence of neuro-ophthalmologic symptoms, median age of onset of the disease, and patient sex in different leukodystrophy forms in the HLC and LLC.

			Sex					Sex *	
Disease	*n*/*n* HLC (%)	Median AO in Months	m	f	Disease	*n*/*n* LLC (%) *	Median AO in Months *	m	f
Total	148/217 (68%)	7	128	89	Total	831/1090 (76%)	6	655	219
MLD	21/50 (42%)	63	26	24	MLD	39/122 (32%)	24	15	12
CD	34/34 (100%)	2	17	17	CD	29/35 (83%)	7	21	11
KD	14/25 (56%)	5	12	13	KD	47/86 (55%)	11	37	31
X-ALD	13/22 (59%)	94	22	0	X-ALD	32/47 (68%)	83	61	0
PMD	13/13 (100%)	1	13	0	PMD	285/291 (98%)	2	254	11
VWM	7/12 (58%)	25	6	6	VWM	7/24 (29%)	30	8	13
AxD	3/10 (30%)	13	5	5	AxD	4/6 (67%)	8	2	3
AGS	6/9 (67%)	5	6	3	AGS	2/3 (67%)	4	3	0
MLC	2/5 (40%)	11	4	1	MLC	1/1 (100%)	5	1	0
Others	27/37 (73%)	4	17	20	others	385/475 (81%)	3	253	138

HLC = Hamburg leukodystrophy cohort, LLC = literature leukodystrophy cohort, AO = age of onset of the disease, MLD = metachromatic leukodystrophy, CD = Canavan disease, KD = Krabbe disease, X-ALD = X-linked adrenoleukodystrophy, PMD = Pelizaeus–Merzbacher disease, VWM = vanishing white matter disease, AxD = Alexander disease, AGS = Aicardi–Goutières syndrome, MLC = megalencephalic leukoencephalopathy with subcortical cysts, others = less frequent leukodystrophies. * Clinical data on the occurrence of neuro-ophthalmologic symptoms could be determined for 1090/1153 patients. Clinical data on sex could be determined for 874/1153 patients. Clinical data on age of onset of the disease could be determined for 654/1153 patients.

**Table 2:** Frequency of different neuro-ophthalmologic symptoms in leukodystrophy patients of the HLC and LLC related to the total number of pathologic findings.

	*n*/*n* HLC (%)	*n*/*n* LLC (%)
Total NOS	366	1057
EMD	111/366 (30%)	637/1057 (60%)
Fixational eye movement	55/366 (15%)	24/1057 (2%)
Posterior segment findings	47/366 (13%)	145/1057 (18%)
Refractive errors	37/366 (10%)	not specified
Pathological light reflex	36/366 (10%)	28/1057 (3%)
Visual function	23/366 (6%)	223/1057 (21%)
Anterior and periocular segment findings	23/366 (6%)	not specified

HLC = Hamburg leukodystrophy cohort, LLC = literature leukodystrophy cohort, NOS = neuro-ophthalmologic symptom, EMD = eye movement disorders.

**Table 3:** Occurrence of specific neuro-ophthalmologic symptoms in absolute numbers and percentages in different leukodystrophies in the HLC and LLC.

	Nystagmus (EMD)		Strabismus (EMD)		FEMD		Visual Function		Pathological Light Reflex	
	*n*/*n* HLC (%)	*n*/*n* LLC (%)	*n*/*n* HLC (%)	*n*/*n* LLC (%)	*n*/*n* HLC (%)	*n*/*n* LLC (%)	*n*/*n* HLC (%)	*n*/*n* LLC (%)	*n*/*n* HLC (%)	*n*/*n* LLC (%)
Total	60/217 (28%)	569/790 (72%)	40/217 (18%)	68/202 (34%)	55/217 (25%)	24/35 (69%)	23/217 (11%)	223/417 (53%)	31/217 (14%)	28/72 (39%)
PMD	13/13 (100%)	273/276 (99%)	4/13 (31%)	3/11 (27%)	2/13 (15%)	1/1 (100%)	0/13 (0%)	24/94 (26%)	1/13 (8%)	not specified
CD	21/34 (62%)	9/15 (60%)	9/34 (26%)	not specified	19/34 (56%)	7/17 (41%)	7/34 (21%)	14/14 (100%)	7/34 (21%)	1/1 (100%)
VWM	3/12 (25%)	1/1 (100%)	2/12 (17%)	not specified	2/12 (17%)	not specified	1/12 (8%)	1/1 (100%)	1/12 (8%)	1/1 (100%)
AGS	2/9 (22%)	2/3 (67%)	2/9 (22%)	not specified	4/9 (44%)	1/1 (100%)	0/9 (0%)	not specified	3/9 (33%)	not specified
AxD	2/10 (20%)	4/4 (100%)	0/10 (0%)	1/1 (100%)	0/10 (0%)	not specified	0/10 (0%)	not specified	1/10 (10%)	not specified
KD	4/25 (16%)	13/112 (12%)	3/25 (12%)	5/35 (14%)	7/25 (28%)	2/2 (100%)	2/25 (8%)	59/108 (44%)	4/25 (16%)	20/34 (59%)
X-ALD	1/22 (5%)	4/4 (100%)	5/22 (23%)	19/43 (44%)	6/22 (27%)	not specified	6/22 (27%)	36/57 (63%)	1/22 (5%)	not specified
MLD	2/50 (4%)	3/3 (100%)	5/50 (10%)	17/63 (27%)	7/50 (14%)	not specified	1/50 (2%)	not specified	11/50 (22%)	not specified
MLC	0/5 (0%)	not specified	0/5 (0%)	not specified	0/5 (0%)	not specified	0/5 (0%)	not specified	0/5 (0%)	not specified
others	12/37 (32%)	260/372 (70%)	10/37 (27%)	23/49 (47%)	7/37 (19%)	13/14 (93%)	6/37 (16%)	89/143 (63%)	2/37 (5%)	6/36 (17%)

HLC = Hamburg leukodystrophy cohort, LLC = literature leukodystrophy cohort, EMD = eye movement disorder, FEMD = fixational deficit, MLD = metachromatic leukodystrophy, CD = Canavan disease, KD = Krabbe disease, X-ALD = X-linked adrenoleukodystrophy, PMD = Pelizaeus–Merzbacher disease, VWM = vanishing white matter disease, AxD = Alexander disease, AGS = Aicardi–Goutières syndrome, MLC = megalencephalic leukoencephalopathy with subcortical cysts, others = less frequent leukodystrophies.

**Table 4:** Fraction of pathological VEP findings in patients with different leukodystrophies.

	*n*/*n* HLC (%)	*n*/*n* LLC (%)
Total	35/54 (65%)	52/79 (66%)
PMD	4/4 (100%)	13/16 (81%)
AGS	1/1 (100%)	not specified
MLC	2/2 (100%)	1/1 (100%)
CD	4/5 (80%)	1/3 (33%)
MLD	11/19 (58%)	9/12 (75%)
VWM	2/4 (50%)	not specified
X-ALD	2/5 (40%)	3/5 (60%)
AxD	1/3 (33%)	0/2 (0%)
KD	1/4 (25%)	23/37 (62%)
others	7/7 (100%)	2/3 (66%)

HLC = Hamburg leukodystrophy cohort, LLC = literature leukodystrophy cohort, PMD = Pelizaeus–Merzbacher disease, AGS = Aicardi–Goutières syndrome, MLC = megalencephalic leukoencephalopathy with subcortical cysts, CD = Canavan disease, MLD = metachromatic leukodystrophy, VWM = vanishing white matter disease, X-ALD = X-linked adrenoleukodystrophy, AxD = Alexander disease, KD = Krabbe disease, others = less frequent leukodystrophies.

## Data Availability

The data presented in this study are not available in a more detailed form due to data protection reasons.

## Supplementary Materials

The following supporting information can be downloaded at: https://www.mdpi.com/article/10.3390/jcm13175114/s1, Table S1: Optic atrophy in the literature cohort.
