# Supplementary material for: Overview of Neuro-Ophthalmic Findings in Leukodystrophies
Source: J Clin Med. 2024 Aug 28;13(17):5114. doi: 10.3390/jcm13175114 (PMC11396446; doi:10.3390/jcm13175114)
Supplement: Supplementary file 1 [file jcm-13-05114-s001.zip › jcm-3123329-Supplementary Materials.pdf]

Supplementary Materials

Table S1. Occurrence of optic atrophy in absolute numbers and percentage in different leukodystrophies in HLC and LLC.

	n/n HLC (%)	n/n LLC (%)
**total**	**30/217 (28%)**	**145/385 (38%)**
**PMD**	4/13 (31%)	2/5 (40%)
**CD**	5/34 (15%)	11/30 (37%)
**VWM**	2/12 (17%)	6/23 (26%)
**AGS**	3/9 (33%)	not specified
**AxD**	0/10 (0%)	not specified
**KD**	5/25 (20%)	33/128 (28%)
**X-ALD**	2/22 (9%)	12/21 (57%)
**MLD**	1/50 (2%)	12/46 (26%)
**MLC**	0/5 (0%)	not specified
**others**	8/37 (22%)	69/132 (52%)

LLC=literature leukodystrophy cohort, AGS=Aicardi-Goutieres-Syndrom, CD=Canavan disease, KD=Krabbe disease, MLD=metachromatic leukodystrophy, PMD=Pelizaeus-Merzbacher disease, VWM=vanishing white matter disease, X-ALD=X-linked adrenoleukodystrophy, others=less frequent leukodystrophies.
